# Cadmium and Lead in Blood Cockle (*Anadara granosa*) from Asajaya, Sarawak, Malaysia

**DOI:** 10.1155/2014/924360

**Published:** 2014-11-04

**Authors:** Md. Faruk Hossen, Sinin Hamdan, Md. Rezaur Rahman

**Affiliations:** Faculty of Engineering, Universiti Malaysia Sarawak, 94300 Kota Samarahan, Sarawak, Malaysia

## Abstract

The concentrations were ranged from 1.35 ± 0.16 to 2.22 ± 0.34 *µ*g/g (dry weight) and 2.65 ± 0.34 to 4.36 ± 0.53 *µ*g/g (dry weight) for Cd and Pb, respectively, in blood cockle *Anadara granosa* from four sites of Sabang River, namely, Kampung Sambir, Kampung Tambirat, Beliong Temple, and Kampung Tanjung Apong, which are located at Asajaya, Sarawak, Malaysia. All values exceeded safety limits set by Malaysian Food Regulation (1985). It may be the cause of serious human health problems after long term consumption. Thus, consumer should have consciousness about such type of seafood from mentioned sites and need further investigation.

## 1. Introduction

The blood cockle* Anadara granosa* is a bivalve mollusc in the family Arcidae and subfamily Anadarinae and locally known as “kerang” in Malaysia [1]. The bivalves in this family are renowned for a source of cheap protein in tropical areas, especially in the Indo-Pacific region [2]. Therefore, marine bivalve particularly* A. granosa* is of considerable economic importance in Malaysia [3]. Since* A. granosa* is a filter feeding organism, contamination of the highly productive mudflats with heavy metals tends to be accumulated in their whole body tissue. This could serve as an important environmental sink of heavy metals [4] and provide an indication of river pollution.

Sarawak like West Malaysia is presently undergoing rapid industrial development and there have been incidences of toxic pollution from industry [5–7]. Asajaya is a small Sarawakian town located in the Samarahan Division and adjacent to industrial areas which were reclaimed from mangrove. The types of industry in this area presently in operation include food processing and canning, processing of agricultural products, feed mills, timber based wood products, and transport equipment. Sabang is a main river flowing into mentioned area where some fishing villages are situated and connected with Sarawak River and South China Sea. Seafood such as cockles is supplied from Asajaya to most of the seafood markets in this division. Solid and liquid wastes emanating from the industrial activities are the inevitable byproducts of manufacturing process. These wastes contain toxic chemicals and other substances including toxic heavy metals [8]. A number of natural and anthropogenic sources produce heavy metals. Heavy metals such as Cd and Pb are toxic even at relatively low concentration and not essential for metabolic activities [9, 10]. The abundance of heavy metal may jeopardize human health due to the consumption of contaminated bivalves [11]. For examples, Cd may cause human carcinogen; Pb can damage blood circulation [12–14].

People are becoming more aware of the complexity of the nature and the delicate balance that exist within the global ecosystem [15]. The discharge of effluents and associated toxic compounds into aquatic systems represents an ongoing environmental problem due to their possible impact on communities in the receiving aquatic water and a potential effect on human health [16]. In particular, in highly polluted and industrial areas, point and nonpoint sources of anthropogenic chemicals and metals have polluted rivers with highly complex mixtures of chemicals and other anthropogenic perturbations to degree where life in rivers is severely impacted [17]. Therefore, the objective of this study has been to determine the concentrations of two toxic metals (Cd and Pb) in blood cockles sampled from four different sites near Sabang River of Asajaya.

## 2. Method and Materials 

### 2.1. Description of Study Sites

**Figure 1:**
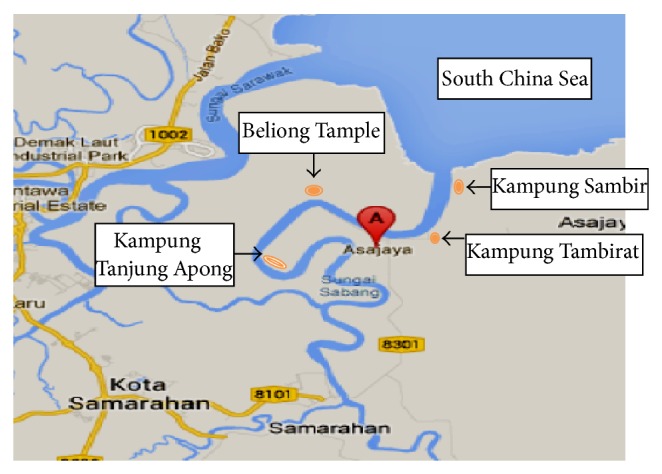
Map of Asajaya showing sampling sites.

The blood cockles* Anadara granosa* were collected during May 14 from four sites of Sabang River, namely, Kampung Sambir, Kampung Tambirat, Beliong Temple, and Kampung Tanjung Apong, which are located at Asajaya, Sarawak, Malaysia, as shown in Figure 1. Sabang is a main river flowing into mentioned area where some fishing villages are situated. This river is connected with Sarawak River and with South China Sea. The sites are located adjacent to industrial areas which were reclaimed from mangrove. Currently, the types of industries are food processing and canning, processing of agricultural products, feed mills, timber based wood products, and transport equipment.

### 2.2. Sampling and Metal Analysis

Cockles were transported, refrigerated at 4°C, to the laboratory within 4 h of collection. After a preliminary shell clean-up, the specimens were frozen and maintained at −18°C pending processing. For analysis, all individuals from one site were defrosted; the shells were carefully removed using Teflon-covered forceps and a stainless steel surgical blade; the soft tissues were then freeze-dried at −80°C for about 24 h. These dried samples were then ground to obtain a homogenous powder and stored separately in acid-cleaned glass bottles in desiccators at room temperature until analysis. Approximately 0.5 g of the samples was digested with 10 mL of 65% analytical grade HNO_3_ into a microwave digestion vessel and it was then capped tightly. The digestion vessel (bomb) was then placed in a normal, conventional microwave oven and cooked for two minutes at a 40% power level. The vessel was then cooled for five minutes and then recooked for four minutes at 30% power level. The completely digested sample was then allowed to cool until the fumes dissipated. The inner side of the cap was then cleaned with distilled water and the washed liquid was added to the original sample mixture in the vessel and then it was subjected to filtration using number #44 Whatman filter paper. The sample solution was diluted to a 50 mL graduated volumetric flask and then subjected to metal determination.

Flame atomic absorption spectrometer (FAAS; Perkin-Elmer HGA-600) was employed for the analysis of cadmium and lead. Standard mixtures of 0.05, 0.1, 0.2, 0.5, and 1.0 ppm of Cd and Pb were prepared in nitric acid solution for calibration. The accuracy of the methods was assessed using certified reference material DORM-2 in triplicate, and mean recovery was 95.5 ± 1.3% for both metals. All data were expressed on a dry weight basis (*μ*g/g dry wt.).

## 3. Results and Discussion

It was observed that the concentrations ranges were 1.35 ± 0.16 to 2.22 ± 0.34 *μ*g/g (dry weight) and 2.65 ± 0.34 to 4.36 ± 0.53 *μ*g/g (dry weight) for Cd and Pb, respectively, in cockles (Table 1). The highest values (2.22 ± 0.34 *μ*g/g for Cd and 4.36 ± 0.53 *μ*g/g for Pb) were observed in specimens from Kampung Tanjung Apong which is located near the connecting point of Sabang River and Sarawak River and the second highest values (1.85 ± 0.28 *μ*g/g for Cd and 3.55 ± 0.48 *μ*g/g for Pb) were observed from Kampung Sambir which is located near the connecting point of Sabang River and China Sea. The levels of Cd and Pb from studied sites exceeded the maximum permissible limits set by Malaysian Food Regulations (1985).

The highest value of Cd may be due to the influence of external discrete sources like industrial activities, agriculture runoff and other anthropogenic inputs [11, 18] and sites are within the vicinity of agricultural areas mostly large oil palm plantations heavy in pesticides and herbicides used [5, 19]. After long term consumption, this may be the cause of possible toxicological risks and heavy metal related diseases, such as Parkinson's and Wilson's diseases [20]. On the other hand, the highest value of Pb may result from burning of fossil fuels from boats used for fishing and also leisure activities [6, 21]. This may be the cause of neurological deficits such as mental retardation in children and kidney disease such as interstitial nephritis to adults and also contribute to hypertension and cardiovascular disease [22] to the consumers after long term consumption. Previous study from the northwest coast of Peninsular Malaysia, in the state of Penang, showed that the levels of heavy metals analyzed for cockles* A. granosa* were ranged from 0.87 to 0.89 and 0.11 to 0.12 *μ*g/g dry weights for Cd and Pb, respectively [23]. The results indicate that the levels of Cd and Pb in* A. granosa* from Penang were lower than safety limits set by Malaysian Food Regulations (1985) as well as lower than those obtained in this research findings. Another research revealed that the levels of heavy metals analyzed for green-lipped mussels* Perna viridis* collected along the west coast of Peninsular Malaysia were ranged from 0.68 to 1.25 and 2.51 to 8.76 *μ*g/g dry weights for Cd and Pb, respectively [24]. The concentration of Cd was below the reference values for human consumption set by Malaysian Food Regulations (1985) whereas the concentration of Pb was above the reference values. The results also indicate that the level of Pb in the present investigation was within the value from the west coast of Peninsular Malaysia. The use of coastal waters as a convenient receptacle for domestic and industrial wastes threatens the quality of seafood rivers and coastal waters are presently exposed to not only the increasing quantities of metals and nutrients, but also the cocktails of industrial derived contaminants, many of which exhibit significant persistence and capabilities for bioaccumulation [25].

However, it revealed that the cockles in this research area have been contaminated through Cd and Pb caused by agriculture runoff and industrial and other anthropogenic activities which can be the cause of serious human health problems for long term consumption.

## 4. Conclusion

It is concluded that the concentrations of Cd and Pb in blood cockles from four sites of Asajaya exceeded the maximum permissible limits set by Malaysian Food Regulation (1985). It showed that cockles from the mentioned area have been contaminated through Cd and Pb caused by agriculture runoff and industrial and other anthropogenic activities. It may be the cause of serious human health problems such as Parkinson's and Wilson's diseases and also mental retardation in children and kidney disease such as interstitial nephritis to adults and also contribute to hypertension and cardiovascular disease after long term consumption. Thus, consumer should have consciousness about such type of seafood from mentioned sites and need further investigation.

## Figures and Tables

**Table 1:** The concentrations (*µ*g/g dry wt) of Cd and Pb in blood cockle *Anadara granosa* from four sites of Sabang River at Asajaya, Sarawak, Malaysia.

Sampling sites	Cd (mean ± SD)	Pb (mean ± SD)
Kampung Sambir	1.85 ± 0.28	3.55 ± 0.48
Kampung Tambirat	1.35 ± 0.16	2.65 ± 0.34
Beliong Temple	1.58 ± 0.23	2.94 ± 0.42
Kampung Tanjung Apong	2.22 ± 0.34	4.36 ± 0.53
The maximum permissible limits set by Malaysian Food Regulations (1985)	**1.00**	**2.00**

SD: standard deviation.
